# 

**Published:** 1875-05

**Authors:** 


CONTENTS:

| | PAGE. |
|---|---|
| Nervous Diseases | 133 |
| Dine Early and Eat Less | 134 |
| A Remedy for Vomiting | 136 |
| Self-Inflicted | 137 |
| A Dangerous Practice | 138 |
| Our Summer at Maplewood, Chap. IV., Food and Fancy. | 139 |
| The Contagion of Scarlet Fever | 144 |
| An Omission | 145 |
| Lime in Food | 146 |
| Thermometers | 146 |
| Hash | 147 |
| Making an Invalid | 149 |
| Spider Cancer | 150 |
| Cholera Infantum | 151 |
| Fever and Ague | 153 |
| Inhalation | 154 |
| Strength | 155 |
| The Chief Adornment | 156 |
| The House of Steinway | 157 |
| Don't Take Down the Stove | 158 |
| Our Battle and our Foes | 159 |
| Preserving Food | 160 |
| De Graffe and Cochrane | 161 |
| Shall we Possess our Soles in Comfort | 161 |

